# Benthic Biofilm Bacterial Communities and Their Linkage with Water-Soluble Organic Matter in Effluent Receivers

**DOI:** 10.3390/ijerph19041994

**Published:** 2022-02-10

**Authors:** Longfei Wang, Yutao Wang, Yi Li, Wenlong Zhang, Huanjun Zhang, Lihua Niu, Nuzahat Habibul

**Affiliations:** 1 Key Laboratory of Integrated Regulation and Resource Development on Shallow Lakes, Ministry of Education, College of Environment, Hohai University, Nanjing 210098, China; lfwang@hhu.edu.cn (L.W.); yubao15@163.com (Y.W.); 1223zhangwenlong@163.com (W.Z.); zhanghuanjun@hhu.edu.cn (H.Z.); niulh@hhu.edu.cn (L.N.); 2 College of Chemistry and Chemical Engineering, Xinjiang Normal University, Urumqi 830054, China; nuzahat@163.com

**Keywords:** biofilm resilience, water-soluble organic matter, microbial community, co-occurrence network

## Abstract

Benthic biofilms are pioneering microbial aggregates responding to effluent discharge from wastewater treatment plants (WWTPs). However, knowledge of the characteristics and linkage of bacterial communities and water-soluble organic matter (WSOM) of benthic biofilms in effluent-receiving rivers remains unknown. Here, we investigated the quality of WSOM and the evolution of bacterial communities in benthic biofilm to evaluate the ecological impacts of effluent discharge on a representative receiving water. Tryptophan-like proteins showed an increased proportion in biofilms collected from the discharge area and downstream from the WWTP, especially in summer. Biofilm WSOM showed weak humic character and strong autochthonous components, and species turnover was proven to be the main factor governing biofilm bacteria community diversity patterns. The bacterial community alpha diversity, interspecies interaction, biological index, and humification index were signally altered in the biofilms from the discharge area, while the values were more similar in biofilms collected upstream and downstream from the WWTP, indicating that both biofilm bacterial communities and WSOM characters have resilience capacities. Although effluent discharge simplified the network pattern of the biofilm bacterial community, its metabolic functional abundance was basically stable. The functional abundance of carbohydrate metabolism and amino acid metabolism in the discharge area increased, and the key modules in the non-random co-occurrence network also verified the important ecological role of carbon metabolism in the effluent-receiving river. The study sheds light on how benthic biofilms respond to effluent discharge from both ecological and material points of view, providing new insights on the feasibility of utilizing benthic biofilms as robust indicators reflecting river ecological health.

## 1. Introduction

The ecological health of urban rivers and internal lakes have been cumulatively affected by anthropogenic activities. A major impact of urbanization is inputs from wastewater treatment plants (WWTPs) [1]. With the increasing scale of treated sewage, effluent discharge has become one of the most important sources of river replenishment. WWTPs release a multitude of nutrients, dissolved organic matter (DOM), and micropollutants, e.g., pharmaceuticals and personal care products [2,3]. Synthetic chemicals and nutrients mix and enter the receiving waterbodies, triggering eutrophication and altering biogeochemical cycling in fluvial ecosystems [4]. Effluent-receiving waterbodies have been regarded as environmental sensitive regions and have attracted intensive research concerns [5].

Effluent discharge has multidimensional impacts on the ecosystem and functioning of receiving waters. Primarily, effluent input alters the trophic level and constituents of DOM molecules [6], whereafter pelagic algae and communities change in response to trophic level variation and affect primary production. The effects of effluent discharge on other biota, including benthic biofilm, macrophytes, and invertebrates, have also been reported [7]. These effects are likely to be governed by hydrological parameters such as wetting/drying alteration [8], seasonal variation [9]. and dilution of effluent discharge [10]. IN the study [11], the impacts of effluent and hydrological stresses on river functioning were investigated, and it was observed that even highly diluted WWTP effluents can affect the structure of the biofilm community and river ecosystem functions.

Benthic biofilms are assemblages of living and dead algae, microbes, and organic debris, constituting the basis of the benthic food web [12,13]. Benthic biofilms have been considered pioneer microbial aggregates in response to effluent discharge [12]. The architecture and functioning of biofilms are constantly changing following variations in dissolved oxygen, organics, hydrodynamics, etc. [14]. The total biomass, microbial composition, photosynthesis of algae, and assimilation of organic matter are accordingly modified as a consequence [15]. Existing studies have shown that benthic biofilms can restrain pollutants, e.g., heavy metals, pharmaceuticals and brominated flame retardants released from historical events and transfer the contaminants to higher trophic levels [16,17]. Hence, biofilms are often used as indicators to evaluate ecological changes in aquatic environments by assessing their respiratory rate [18] or soluble reactive phosphorus uptake capacity [11]. Biofilms adapted to anthropogenic disturbances typically show higher resistance to effluent discharge [4,11]. Water-soluble organic matter (WSOM) is the most active component in biofilm organic matter, comprising carbohydrates, amino acids, and organic acids. Effluent discharge has been verified to promote extracellular enzyme activities, e.g., leucine aminopeptidase and amino glucosidase in benthic biofilms, facilitating the conversion of complex organic matter and its subsequent uptake and utilization by microbes [19,20].

A number of studies have demonstrated the complicated and bidirectional relationship between organic carbon and bacteria communities in rivers. River organic carbon serves as a carbon source and nutrient for heterotrophic bacteria and some algae, and can be metabolized by micro-organisms in the aquatic environment [21]. Different from the organic contents in other matrices, such as sediments, the organic compounds in biofilm may affect the microbial community structure and modify the food web character and energy transfer efficiency [12]. Chromophore DOM (CDOM) is an optically active part in the bulk DOM pool that can intensely absorb light in the ultraviolet and blue spectral regions, and exhibits remarkable changes in the quantity and quality of diverse biogeochemical processes [6]. Avila et al. unveiled the dynamic succession between CDOM and the microbial community in a small river dominated by effluent discharge and confirmed a remarkable response of actinomycetes and protein components [22]. Zhang et al. explored the relationships between phytoplankton communities and CDOM in a tropical lake and found that CDOM could affect bacterial community structure by participating in the metabolism of specific bacterial communities. Similar results were observed by analyzing the connection between phytoplankton community and CDOM in a eutrophic lake [23]. Nonetheless, most studies have focused on the characteristics and association between planktonic communities and DOM in streams, while the bacterial community structure and interspecific interactions of organic compounds in adherent aggregates, e.g., benthic biofilms, have been largely ignored. An analysis linking the microbial community and WSOM components in benthic biofilms may help to bring forward biological indicators for assessing freshwater quality and ecosystem fitness.

In the past decade, the rapid development in bioinformatics has afforded technical support to decipher responses of microbial community assembly and metabolic functions to environmental stresses [23,24]. Burdon et al. confirmed the resilience of microbial communities to effluent discharge and found that the buffering capacity of microbial communities is vulnerable to impact by environmental fluctuations [2]. Recently, co-occurrence network analysis has been employed to explain the interspecific interactions of microbial communities in suspended particulates, soils, and sediments. These studies manifest that microbial communities usually have non-random co-occurrence patterns and a modular structure, implying the vital role of biological interactions in adjusting the fluvial ecosystem functioning [25,26,27]. Unfortunately, no studies have been performed to evaluate microbial interactions in river biofilms in response to effluent discharge; thus, the co-occurrence patterns between bacterial communities and biofilm WSOM have not been elucidated yet.

In this case study, we investigated how benthic biofilm bacterial communities and biofilm WSOM alter in response to effluent discharge in an effluent-receiving river. We hypothesized that the stress of effluent discharge has a considerable impact on the co-occurrence patterns of benthic biofilm bacterial communities, as well as their linkage with WSOM, especially CDOM. We also hypothesized a higher proportion of metabolic functions on account of nutrient and micropollutant inputs in the discharging area. These results will contribute to unraveling the overall impacts of allochthonous inputs such as effluent discharge on river biofilm properties and ecological functions, providing insights in the search for appropriate pollution indicators in effluent impacted areas and revealing the potential of benthic biofilm as an indicator of the ecological response of effluent-receiving rivers.

## 2. Materials and Methods

### 2.1. Study Site, Experimental Design, and Water Characteristics

**Figure 1:**
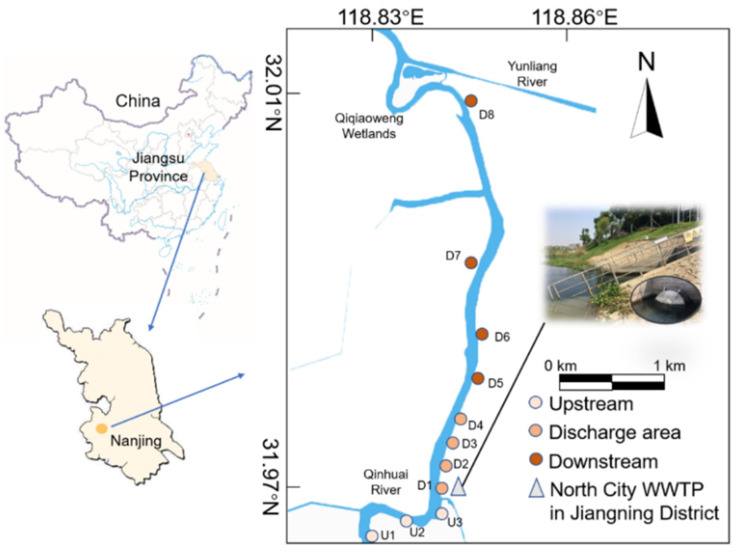
Location of sampling sites in Qinhuai River receiving effluent from North City wastewater treatment plant (WWTP) in Jiangning District, Nanjing, China.

The North City WWTP of Jiangning District (31°58′ N, 118°50′ E) uses an oxidation ditch process that treats the sewage of >40 thousand P.E. from domestic sources. On average, 70 thousand cubic meters of wastewater are treated per day and the effluent is discharged into the Qinhuai River, located in Nanjing, Jiangsu Province, China, as illustrated in Figure 1. The water level and temperature of the Qinhuai River show significant seasonal differences (Table S1). Nearly 75% of the annual precipitation is concentrated during the May-to-September rainy season (summer) [28]. The area of interest has a straight channel with few tributary confluences, and the WWTP effluent discharge is the main external input to the reach, endowing the area with superiority for evaluating the effects of wastewater effluent on ecosystem functioning.

Eleven sampling sites were selected along the reach. The sites were categorized into three areas according to their relative positions to the effluent outfall shown in Figure 1. U1 to U3, 1, 0.5, and 0.2 km upstream from WWTP effluent discharge, are referred to as the Upstream group; D1 to D4, 0, 0.2, 0.5, and 0.7 km downstream the discharge, are referred to as the Discharge area group; D5 to D8, 1, 1.5, 2, 3 km, downstream from the discharge, are referred to as the Downstream group. The values of pH, temperature (T, °C). and dissolved oxygen (DO) were measured using a portable meter (HQ30d, HACH Company, Loveland, CO, USA) at each sampling site in December 2019 and September 2020, respectively. Water and benthic biofilms were collected on 26 December 2019 (winter, averaged water temperature: 10.3 °C) and 8 September 2020 (summer, averaged water temperature: 26.8 °C) (Table S1). Total nitrogen (TN) and total phosphorus (TP) were measured to reflect the trophic conditions at varying sampling sites [29]. Three parallel water samples were collected and analyzed at each site.

### 2.2. Biofilm Harvesting and Water-Soluble Organic Matter (WSOM) Extraction

Benthic biofilms were collected following the protocols reported by Wang et al. [30]. The main steps of collection include: rock selection, biofilm scraping, sample preservation, and transportation. The detailed collection method is available in SI. Prior to extraction, 20 g of biofilm samples in wet weight were lyophilized using a freeze dryer (Christ ALPHA 1-2 LD plus, Marin Christ Co., Osterode am Harz, Germany). To improve the extraction efficiency, we ground the lyophilized biofilm and passed it through a 100-mesh sieve to remove impurities. Biofilm WSOM was extracted using a leaching-centrifugation method according to the protocols of previous studies [31,32]. Pre-treated biofilm was packed into sterilized conical bottles at a material-to-water (g:g) ratio of 1:3. Then the samples were shaken for 16 h at ambient temperature. Afterwards the leachate was transferred to 50 mL sterilized centrifugal tubes and then centrifuged at 4000× *g* r/min for 30 min. The supernatant was filtered through a 0.45 μm sterile acetate membranes, and the generated filtrate was defined as biofilm WSOM and stored at −4 °C prior to analytical approaches.

### 2.3. Spectral Analyses of Biofilm WSOM

Dissolved organic carbon (DOC) concentrations in bulk WSOM solutions were measured using a total organic carbon analyzer (Multi N/C2100, Analytik Jena, Jena, Germany). The DOC concentration in biofilm WSOM were normalized to mg/g shown in Table S2. UV-vis absorption spectra were measured by a spectrophotometer (UV-1800, Shimadzu, Japan). The excitation–emission matrices (EEMs) of CDOM in WSOM were measured using a fluorescence spectrophotometer (F7000, Hitachi, Japan). Detailed information on spectral analysis is provided in SI. Parallel factor analysis (PARAFAC) was performed using the DOM Fluor toolbox in MATLAB (R2017a) software [33]. The relative contents of fluorescent components were obtained via F_max_ values analyzed by PARAFAC. Several UV-visible spectra-derived parameters were calculated to demonstrate the aromaticity (SUVA_254_), hydrophobicity (E_254_/E_204_), and molecular weight (S_R_) of biofilm WSOM. We also calculated parameters including biological index (BIX), humification index (HIX), and fluorescence index (FI) to describe the fluorescent characteristics of biofilm WSOM as described in the Table S3.

### 2.4. Microbial Community Analysis

#### 2.4.1. DNA Extraction and PCR Amplification

For each biofilm sample, DNA extraction was executed through the E.Z.N.A.^®^ Soil DNAKit (Omega Bio-tek, Norcross, GA, USA), following the manufacturer’s instructions. Agarose gel electrophoresis was used to analyze the quality of the extracted DNA. 16S rRNA gene amplification and Illumina MiSeq sequencing were performed at Biozeron Science and Technology Ltd. (Shanghai, China). The bacterial primers 341F (5′-CCTAYGGGRBGCASCAG-3′) and 806R (5′-GGACTACNNGGGTATCTAAT-3′) were used to amplify the V3−V4 regions of the bacterial 16S rRNA gene. The PCR conditions were as follows: DNA denaturation for 5 min at 95 °C with 27 cycles, at 95 °C for 30 s, 55 °C for 30 s, and 72 °C for 45 s, with a final extension of 10 min at 72 °C.

#### 2.4.2. Sequence Analyses and Functional Prediction

Sequence analyses were carried out via Quantitative Insights Into Microbial Ecology version 2 (QIIME2 version 18.6) software [34]. A single-end sequence data denoising method called the Divisive Amplicon Denoising Algorithm program (DADA2, v1.10) was implemented for processing valid data from BIOZERON Co., Ltd. (Shanghai, China) [35]. We then used the ‘classify-sklearn’ option to assign classification identities to these representative sequences via the ‘qiime feature-classifier’ command, referred to here as Amplicon Sequence Variants (ASVs). These ASVs have more than 99% similarity to the SILVA128 reference comparison database used in classification identities. Information on the sequence reads corresponding to each sample has been uploaded to the NCBI SRA database for public access (bioengineering number: PRJNA717165). Prediction of potential microbial function was performed by an improved metagenome inference method of PICRUSt [36]. Functional gene predictions were performed based on the Kyoto Encyclopedia of Genes and Genomes (KEGG) database. The Nearest Sequenced Taxon Index (NSTI) was used to evaluate the prediction accuracy of PICRUSt, with lower values indicating higher prediction accuracy.

### 2.5. Statistical Analyses

The richness, Pielou, and Shannon indices were calculated in R (version 3.6.2) using the vegan and picante packages. Non-metric multidimensional scaling (NMDS) based on Bray–Curtis distance was performed to decipher the clustering of benthic biofilm bacterial communities among different groups, together with non-parametric multivariate analysis of variance (Adonis) analyzing the significant differences of microbial communities. The dissimilarity indices including the Sørensen dissimilarity index (β_SOR_), the Simpson dissimilarity index (β_SIM_), and the nestedness resultant dissimilarity index (β_NES_) of benthic biofilm bacterial communities were calculated in R, employing the function ‘beta-multi.R’ [37]. The null model was used to quantify the contribution of ecological processes to the microbial assemblage by vegan, ape, and picante packages [38,39].

One-way ANOVA and Tukey’s post hoc test were performed to uncover the differences between groups using SPSS v26 software. STAMP (v.2.1.3) software was used to perform a two-sided Welch’s *t*-test on the functional abundance map predicted by KEGG to discover the metabolic pathways with significant differences between groups. The ‘ggcor’ package was applied to test the correlation between microbial community and spectral indicators via mantel analysis. To integrate spectral information and biological data in the biofilm, redundancy analysis (RDA) was performed using the vegan package in R.

Molecular ecological networks (MENs) corresponding to different seasons were constructed to elucidate the correlation between CDOM fluorophores and bacterial communities using an online MENA pipeline based on a Random Matrix Theory (RMT) bioinformatics approach. To reduce the network complexity, we only selected ASVs that are present in all samples of the same group for network construction. A random network of 100 ASVs corresponding to each empirical network was built to test the statistical significance of the empirical networks [40,41]. The details of network construction are referred to in [42]. Gephi was applied for analyzing network visualization and modularity.

## 3. Results

### 3.1. Spectral Characteristics of Biofilm WSOM

Table S2 summarizes the organic carbon concentrations in biofilm WSOM at varying sampling sites. The normalized DOC concentration of biofilm WSOM ranged from 141.4 to 360.4 mg C/g. The averaged DOC concentration of biofilm WOSM in the Upstream group, Discharge area group, and Downstream group were 175.5, 223.9, and 296.3 mg C/g, respectively. In terms of the UV-visible spectra-derived parameters, the averaged values of SUVA_254_ and S_R_ were 2.96 and 1.91 in winter and were 0.52 and 1.27 in summer, respectively, with significant seasonal differences (*p* < 0.05 and *p* < 0.001) (Table S4).

**Figure 2:**
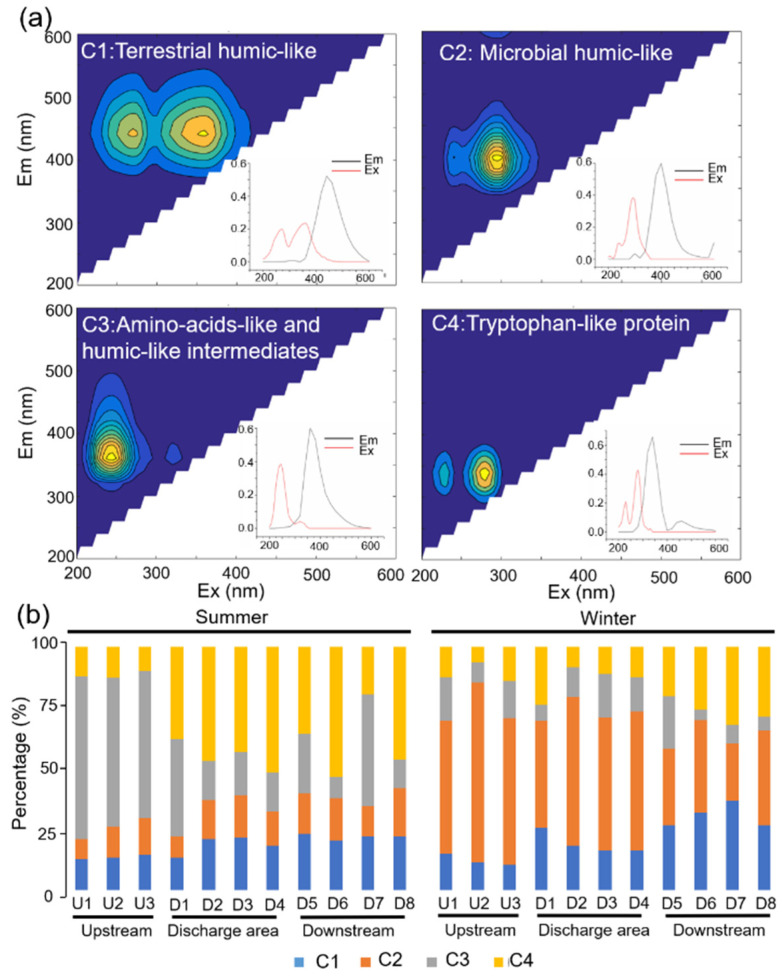
(**a**) Four main components of the spectrum identified by parallel factor analysis (PARAFAC). Insets visualize the excitation and emission loadings of the four components and (**b**) the relative percentages of each component based on F_max_ value.

PARAFAC modeling can identify and verify four fluorescent components, providing a total of 95.72% variability within the data (Figure 2a). The model was compared with available models in the Openflour database, finding a 95% similarity. We found that C1 presented characteristics such as terrestrial humic-like fraction, with high-molecular-weight and photo-labile character [43,44]. C2 also exhibits humus-like properties associated with microbial activity and can be reprocessed in situ by the microbial community [45]. C3 was classified as an intermediate, between humic-like and amino acid-like moiety [46]. C4 serves as a tryptophan-like protein material associated with microbial activity or wastewater discharge [47]. The variation of the percentage of each fluorescent component is shown in Figure 2b. In winter, humic-like fraction (C1 and C2) dominantly accounted for 70% of the total fluorescent components, while in summer, the Upstream group was dominated by humic-like and amino acid-like intermediates (63%), and the Discharge area and Downstream groups were dominated by tryptophan-like proteins (45% and 39%). The percentage of tryptophan-like proteins (C4) in the three areas was 12%, 45%, and 39% in winter and 11%, 14%, and 27% in summer, respectively.

**Figure 3:**
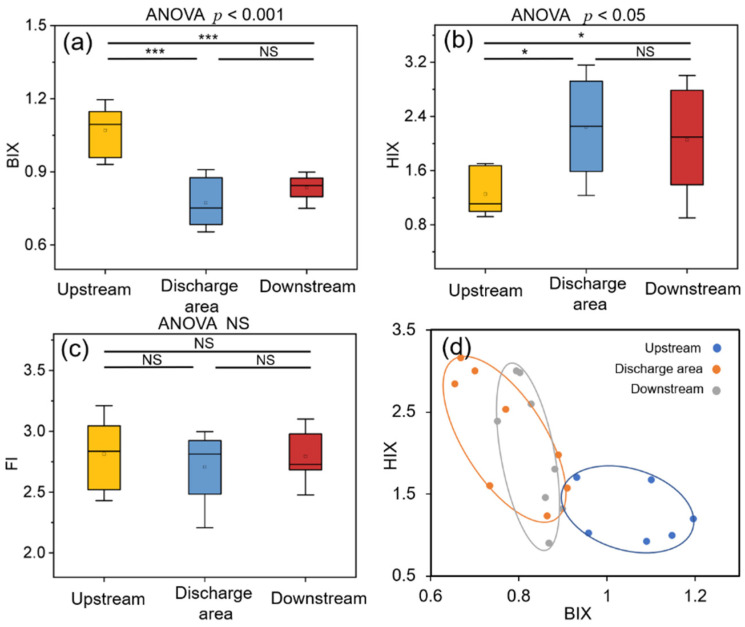
(**a**–**c**) Fluorescence parameters of biofilm water-soluble organic matter (WSOM). Asterisks represent the difference between two groups (* *p* < 0.05; *** *p* < 0.001; NS represents no significant difference) and *p*-values represent the overall group difference. (**d**) Values of biological index (BIX) and humification index (HIX) of biofilm WSOM in different areas: different colors represent different areas, and the horizontal and vertical coordinates indicate the values of BIX and HIX, respectively.

The fluorescent characters of biofilm WSOM exhibit different variations in response to effluent discharge shown in Figure 3a–c. BIX and HIX are significantly different among three areas (*p* < 0.001 and *p* < 0.05), but FI is not. In addition, we found that there was a distinct boundary of BIX-HIX values among three areas (Figure 3d), with the Upstream group clearly isolated from the other two areas, while overlapping was observed between the Discharge area and Downstream groups.

### 3.2. Dynamics, Diversity and Assembly Mechanisms of Bacterial Communities

**Figure 4:**
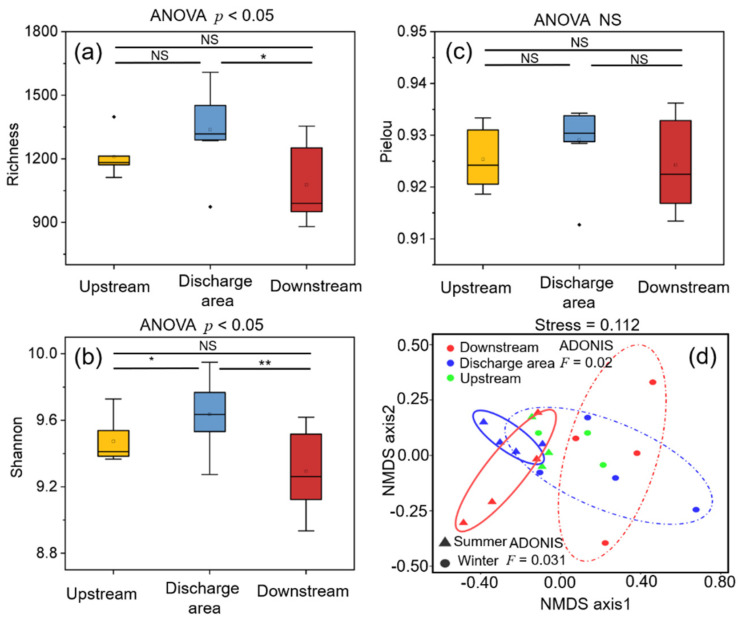
(**a**–**c**) Diversity of bacterial communities and mechanisms of assembly. Comparison of diversity indices in different areas. Asterisks represent the difference between two groups (* *p* < 0.05; ** *p* < 0.001; NS represents no significant difference) and *p*-values represent the overall group difference. (**d**) Non-metric multidimensional scaling (NMDS) analysis by areas and seasons; different colors represent different areas and different shapes represent different seasons.

Figure S1 illustrates the taxonomic composition and relative abundance of bacterial communities at the phylum level in biofilm collected in varying seasons. The results show that Proteobacteria (42.7–65.9%), Actinobacteria (4.8–16.1%), Bacteroidetes (5.2–19.0%) and Chloroflexi (3.6–12.6%) represent the dominant phyla. The microbial α-diversity aspects in Figure 4a,b indicate that the Richness and Shannon indexes are remarkably different among the Upstream, Discharge area, and Downstream groups (*p* < 0.05), with the highest values in the Discharge area group. The averaged Peilou index was highest in the Discharge area group, with no remarkable difference observed among the three areas (Figure 4c). NMDS analysis presented the differences in bacterial communities grouped by sampling time (Figure 4d). Additionally, the Adonis analysis exhibited statistically significant differences in bacterial communities among sampling areas (*F* = 0.02) and between different seasons (*F* = 0.031).

The process of biodiversity change was clarified via two patterns of biome beta diversity: nestedness and turnover. The discrepancy indices of bacterial communities grouped by sampling area and season are shown in Table S5. The mean β_SOR_ value among the three areas was 0.82, with a strong contribution of spatial turnover (β_SIM_ = 0.79) and a small contribution of nestedness (β_NES_ = 0.02). Similar results were also observed for samples grouped by season. We subjected the samples from the Discharge area group and the Downstream group to a resampling procedure in which 100 random samples were taken from six inventories and the mean value β value was calculated, so that the different number of samples from different areas (eight vs. six) was comparable (Table S6). β’_SOR_ in the Upstream group (0.76) is lower than that in the Discharge area group (0.81) and the Downstream group (0.82), which is mainly systematic in the difference of β’_SIM_, while β’_NES_ is almost constant (Table S6). Additionally, we quantified the relative contribution of each microbial ecological process in the assembly among seasons (Figure S2a) and areas (Figure S2b). The ecological processes include homogeneous selection, variable selection, dispersal limitation, homogenizing dispersal, and ecological drift. In winter, variable selection (45.5%) and homogeneous selection (52.7%) accounted for a comparable proportion, while in summer there was lower variable selection (27.3%) and higher homogeneous selection (69.1%). In the grouping by area, homogeneous selection was the dominant factor driving the assembly of bacterial communities in the Upstream group (73.3%) and the Discharge area group (78.6%), whilst in the Downstream group, variable selection (46.4%) and homogeneous selection (50.0%) performed comparably.

### 3.3. Functional Prediction of Bacterial Communities

**Figure 5:**
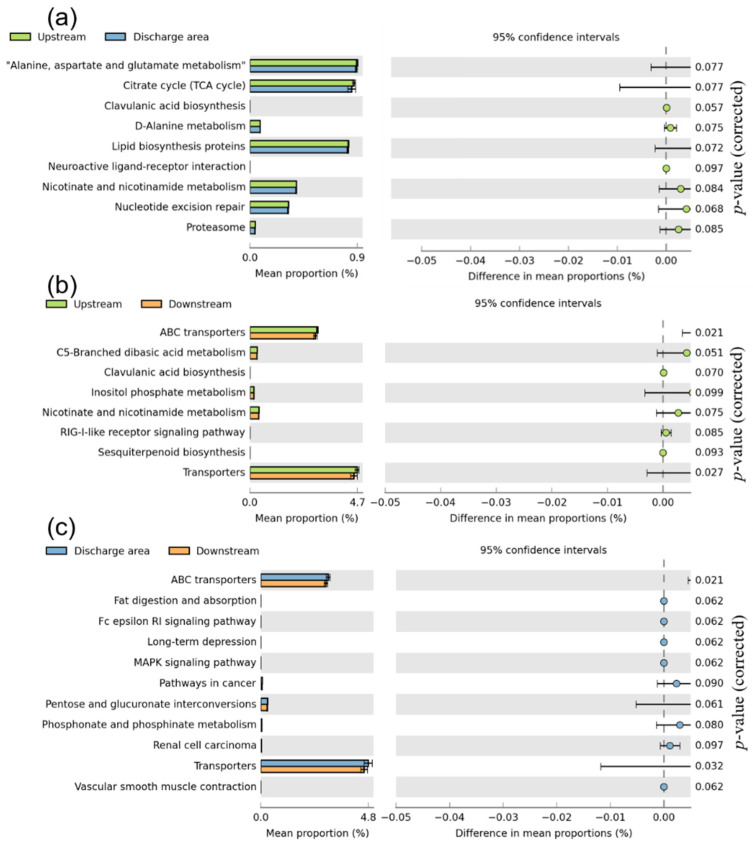
Significant differences in bacterial function predicted by PICRUSt within Kyoto Encyclopedia of Genes and Genomes (KEGG) grouped into level-3 functional categories between (**a**) the Upstream group and Discharge area group, (**b**) the Discharge area group and Downstream group, (**c**) the Discharge area group and Downstream group, using the response ratio method at a 95% confidence interval.

The average NSTI value of all samples was 0.18, indicating that these samples provided an appropriate data set for accurate predictions. By comparing the abundance of KEGG categories predicted by PICRUSt in level-2 metabolic pathways, significant functional differences in distinct sampling areas could be observed (Figure S3). Amino acid metabolism, carbohydrate metabolism, and membrane transport were the three predicted functions with the highest abundance in benthic biofilms, and the values were higher in the Discharge area group than in the groups collected upstream and downstream. We performed a two-by-two comparison of the predicted functions in metabolic pathways at level-3 and discovered that the carbohydrate metabolism (TCA cycle, C5-branched dibasic acid metabolism, and inositol phosphate metabolism), biosynthesis of other secondary metabolites (clavulanic acid biosynthesis), and metabolism of cofactors and vitamins (nicotinate and nicotinamide metabolism) in the Upstream group were significantly different from those in the Discharge area group and the Downstream group (*p* < 0.05, Figure 5a–c). In contrast, only the amino acid metabolism (phosphonate and phosphinate metabolism) was significantly different between the Discharge area group and the Downstream group.

### 3.4. Key WSOM Parameters Affecting Bacterial Community Composition 

**Figure 6:**
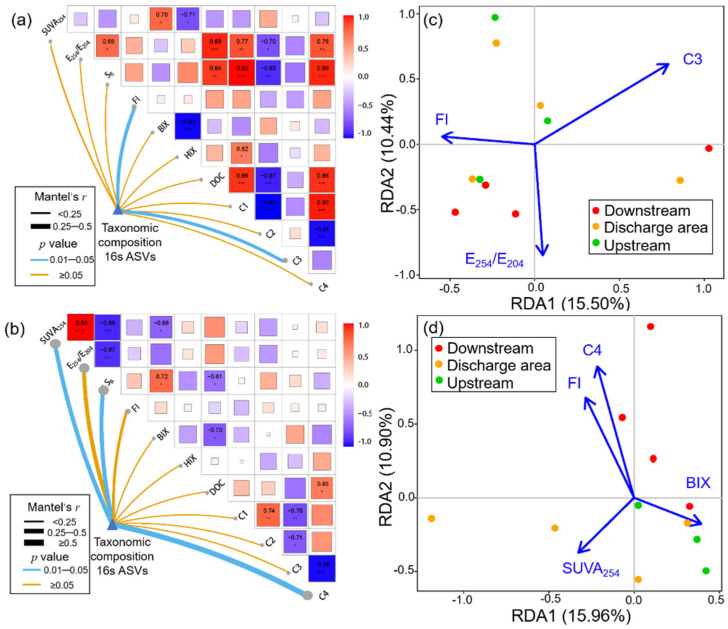
WSOM parameters driving the composition of bacterial communities in the effluent-receiving waterbodies. Mantel test between taxonomic community and biofilm WOSM parameters in (**a**) winter and (**c**) summer. RDA analysis based on bacterial community composition in (**b**) winter and (**d**) summer. (* *p* < 0.05; ** *p* < 0.01; *** *p* < 0.001).

Mantel and RDA analysis were employed to determine the association of biofilm WSOM parameters with bacterial community composition. As shown in Figure 6a, WSOM parameters show more diverse and remarkable correlations with taxonomic compositions in winter. FI and C3 have been confirmed as key factors affecting the composition of bacterial communities in winter (*p* < 0.05), while in summer the key factors are SUVA_254_, S_R_, and C4 (*p* < 0.05, Figure 6b). RDA analysis manifested similar results, as shown in Figure 6c,d. 

The results of the both RDA models proved to be significant (*p* < 0.05) based on the screening of VIF < 5. In winter, humic-like and amino acid-like intermediates (C3) posed the greatest influence on bacterial community composition, while in summer, tryptophan-like proteins (C4) exhibited the strongest impact. We also found that FI was a common key factor influencing the composition of bacterial communities in both seasons.

### 3.5. Co-Occurrence Network Analysis 

**Figure 7:**
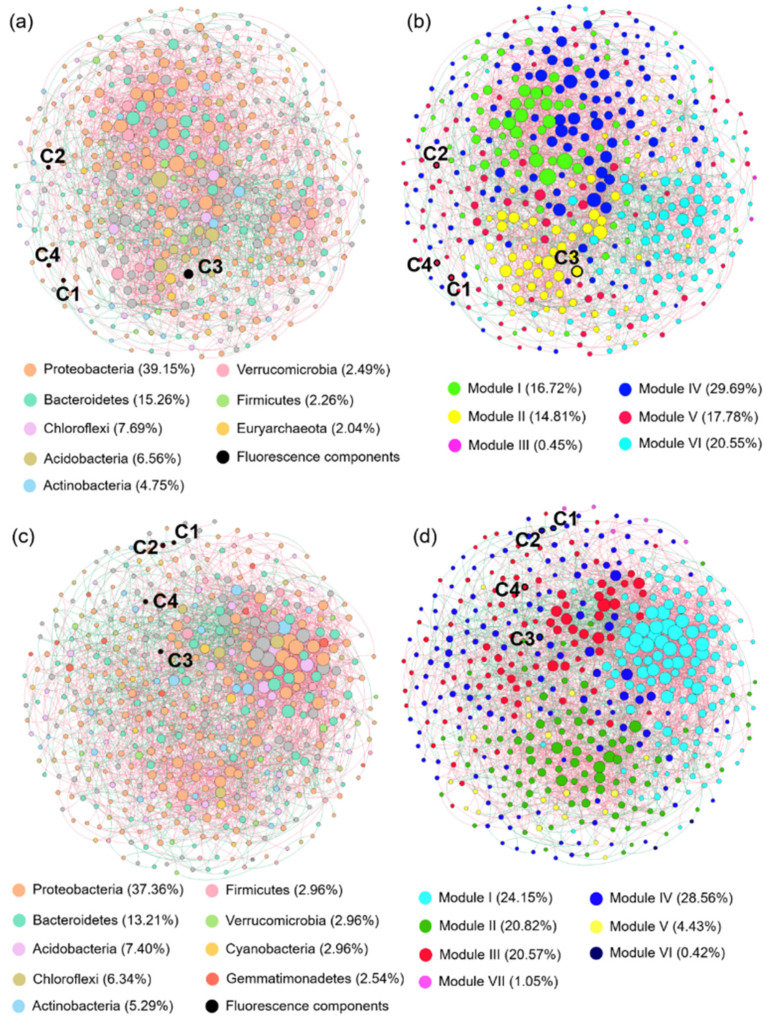
Co-occurrence network based on Random Matrix Theory (RMT) model in (**a**,**b**) winter and (**c**,**d**) summer. The size of each node in the network is proportional to the degree of connectivity, and the nodes are colored according to phylum (**a**,**c**) and different types of modularity classes (**b**,**d**), respectively. Red and green edges indicate positive and negative correlations, respectively.

An RMT-based model was employed to analyze the phylogenetic characters and to determine the symbiotic relationship between bacterial communities and fluorophores. A total of 2564 edges and 443 nodes were obtained in the network derived from samples collected in winter, with a 0.515 modularity encompassing six modules (Figure 7a,b). For the network derived from samples collected in summer, 330 nodes and 1692 edges with a modularity of 0.434 was observed encompassing seven modules (Figure 7c,d). Positive correlations prevailed in both networks derived from winter (76%) and summer (74%). The parameters of the topological network and the random network are shown in Table S7, and the reliability and non-randomness of the empirical network structure is verified by comparing it with the random network analysis [48]. In both networks, Proteobacteria, Bacteroidetes, Acidobacteria, and Chloroflexi occupy the dominant nodes in the network (Figure 7a,c), and are also the predominant bacterial phylum in benthic biofilm bacterial composition, as shown in Figure S1.

Modularity reflects the connectivity within and between clusters, and nodes have closer interactions with each other within the module than with nodes in other modules. There are six modules in the winter network, among which C1, C2, and C4 belong to Module IV, and C3 is affiliated with Module III. Among the seven modules in the summer network, C1, C2, and C3 are grouped into Module IV, and C4 belongs to Module III. By integrating the observations in Figure 6 and Figure 7, we speculate that Module II containing C3 in winter, and Module III comprising C4 in summer serve as the key modules reflecting the impacts of effluent discharge on benthic biofilms.

## 4. Discussion

### 4.1. Effluent Discharge Alters the Nature of Biofilm WSOM

To date, the response of biofilm WSOM to effluent discharge in receiving waterbodies remains unknown. The content of WSOC in biofilm matrix in the discharge area displayed an increase in both seasons (Table S2), implying that effluent discharge could facilitate carbon storage in benthic biofilms. During conventional wastewater treatment processes, high-molecular-weight and aromatic substances are difficult to degrade and a certain amount will be stored in the effluent and discharged to receiving waterbodies [49]. Combined with the variations of SUVA_254_, E_254_/E_204_, and S_R_, it can be inferred that the effluent discharge changed the nature of benthic biofilm WSOM in the Discharge area and the Downstream groups, whereas there are no clear patterns of changes seasonally nor regionally (Table S4). It is difficult to explain such results within the context of an absence of knowledge about the nature of the effluent; therefore, we speculate that the phenomenon could be ascribed to the fluctuating operating conditions of the WWTPs [6,12,49]. Taken together, the findings confirm that the aromaticity, molecular weight, and hydrophobicity of biofilm WSOM in the Downstream group have difficulty recovering to the original state of the Upstream group after receiving effluent.

It is interesting to observe that the fluorescent properties of the biofilm WSOM exhibited a clear resilience responding to effluent discharge. HIX values were signally higher (*p* < 0.05) and BIX values were significantly lower (*p* < 0.001) in the Discharge area group than in the Downstream group (Figure 3). The effluent discharge increased the humification state and decreased the proportions of autochthonous component in benthic biofilms. Changes in protein-like and aliphatic fractions degraded by micro-organisms have been reported to facilitate the humification processes of organic compounds [50]. Meanwhile, the enzymatic processes during biodegradation may promote the enhancement of condensed aromatic structures or the production of structures with increased conjugation, bringing on an increase in HIX [51]. However, unlike the response of DOM in receiving waterbodies, the BIX values of biofilm WSOM exhibited a significant decrease, implying a decline in the input of autochthonous DOM sources [52]. The distinct change between the BIX values in these two studies can be attributed to the adsorption features of biofilm, implying that the biofilm may store allochthonous DOM from effluents. Although the discharge increased the HIX values while decreasing the BIX values, the biofilm in the effluent-receiving river was found to exhibit weak humic character and strong autochthonous components dominated by microbial metabolism [52]. Interestingly, the changes in BIX and HIX indices in the Downstream group show opposite trends (Figure 3d), suggesting that the benthic biofilm WSOM in the downstream area is inclined to recover to the original state in the absence of effluent discharge.

The fluorescent properties of biofilm WSOM differed significantly in response to seasonal changes. The benthic biofilm CDOM in winter is dominated by humic-like materials, whereas intermediate humic-like and amino acid-like dominate in the Upstream group and tryptophan-like proteins occupy the highest fluorescent fractions in the Discharge area and the Downstream groups in summer. The relatively lower proportion of humic-like fractions might be attributed to the high temperature and humid conditions in summer, during which humic-like substances are easily to be released from biofilm WSOM, and winter provides a perfect ecological niche for micro-organisms to release DOM characterized by native protein-like compounds [53]. The protein components of the effluent entrainment increased the amount of tryptophan-like proteins in the benthic biofilm, and this tendency was more pronounced in the warm season. Previous studies have shown that microbes can change the composition of DOM, and the proportion of humic substances is increased through the conversion of protein-like substances [54]. The higher microbial activity in summer may facilitate the conversion process and favor the release and detachment of humic-like fractions. Meanwhile, tryptophan-like proteins are usually associated with anthropogenic activities [6] and some studies have demonstrated a higher removal efficiency of tryptophan-like proteins by WWTP in warmer condition than in colder ones [55]. It must be pointed out that tryptophan-like compounds still dominate the biofilm WSOM in summer, so we consider that the impacts of effluent discharge on the ecological health of rivers cannot be ignored, especially in warmer seasons.

### 4.2. Response of Biofilm Bacterial Communities to Effluent Discharge

The bacterial diversity indices were found to increase in the discharge area and experienced a decrease downstream (Figure 4), with significant changes in the richness and Shannon indices (*p* < 0.05). Studies regarding the effects of effluent discharge on biofilm bacterial communities gave contradictory results, possibly due to either increasing or decreasing microbial diversity and enzyme activity [5,56,57]. In this study, the Discharge area and the Downstream groups had distinct responses to effluent discharge, reflected by the more vulnerable diversity and abundance of bacterial communities, while the diversity of bacterial communities in the Downstream group approached to the status observed in the Upstream group, implying that microbial ecological reconstruction of bacterial community occurred responding to effluent discharge [4]. Additional evidence on the effects of effluent discharge could be found in the co-occurrence network analysis (Figure S4). The discharge increased the diversity of the biofilm bacterial community, interfered with microbial interactions, and reduced the modularity in the Discharge area group. In the Downstream group, there was a recovery in both bacterial interspecies interaction and network modularity.

Figure 4d shows a specific clustering mode among seasons and sampling areas. Pascual-Benito et al. [57] reported a higher microbial diversity in effluent-receiving rivers under high-flow and low-temperature conditions in winter. However, we describe the results of greater differences in bacterial communities across areas under the influence of effluent discharge than seasonal differences (Figure 4d). Considering the relatively lower value of F (0.02) among sampling areas than among seasons (0.031), the ecological impacts of effluent discharge on biofilms in different areas are worthy of being discussed. The assembly pattern analysis suggests an estimated beta diversity β_SOR_ value of bacterial communities among different areas or seasons, with a strong contribution of spatial turnover and a small contribution of nestedness (Table S5). The results suggest that diversity patterns of biofilm in effluent-receiving rivers are primarily caused by species turnover rather than species loss [58]. Turnover is achieved through migration, attachment, and growth, and dispersal refers to the movement of microorganisms in space, especially those absorbed into the biofilms [59]. Typically, the attachment of planktonic cells to the media surface triggers biofilm formation and fundamentally regulates the microbial assembly process [60]. The higher beta diversity can be explained by more diverse environmental conditions. For example, water flow, turbulence, and bottom landscape topography regulate microbial dispersal and colonization patterns, while also producing microhabitats with distinct stresses and mass transfer efficiencies [13]. The increase in β_SOR_ suggests that effluent-borne microorganisms, DOM, and nutrients may be partially adsorbed into the benthic biofilm, resulting in the formation of more complex microhabitats in the discharge area and the area downstream WWTP.

### 4.3. Variations in Metabolic Functions of Bacterial Communities

Effluent discharge affects the original ecosystem functions of rivers, altering the availability of genes related to the carbon cycle and possibly carrying foreign co-energy genes, bringing unknown pressures to riverine ecosystems [23]. The metabolic function of bacteria communities in effluent-receiving rivers is more stable; however, it is important to note that the predicted functional abundance of carbohydrate metabolism and amino acid metabolism increased in the Discharge area group (Figure S3). This may imply that the benthic biofilm bacterial community has a higher rate of carbon turnover and enhanced utilization of carbon sources in areas directly receiving effluent [61]. The membrane transport function was also proven to exhibit a high abundance in this area, suggesting that bacterial cells had active transporter proteins that can transport organic matter and nutrients to facilitate bacterial metabolic processes [24].

In this study, significant differences were observed between alanine/aspartate/glutamate metabolism and nicotinate/nicotinamide metabolism in Upstream group and Discharge area group (Figure 5). Both metabolic pathways are closely related to the degradation of carbohydrates [62]. The involvement of carbohydrate metabolism, cofactors and vitamin metabolism, and other secondary metabolites was higher in the Discharge area and Downstream groups compared to the Upstream group, indicating that the biodegradation activity was higher in areas less affected by effluent discharge [61]. However, the effluent discharge ultimately enhanced the carbohydrate and amino acid metabolism in the Discharge area group, suggesting that the adverse effects of WWTP discharge on the metabolic functions of the benthic biofilm bacterial community may be limited to specific metabolic functions. Another promising finding was that little difference in the metabolic functions of the bacterial community could be found between the Discharge area and the Downstream groups. The results could be ascribed to the partial adaptation of biofilms in response to effluent discharge.

### 4.4. Roles of Bacteria in Shaping Biofilm WSOM

Here, we found significant seasonal patterns, including commonalities and anisotropies, associated with effluent discharge on biofilm WSOM properties and bacterial community composition. In winter, humic-like and amino acid-like intermediates were significantly correlated with bacterial community composition (Figure 6a,b). C3 is mainly comprised of low-molecular-weight and highly aromatic substances [63]. Existing studies have found that bacterial communities are inclined to utilize low-molecular-weight substances [6], and aromaticity is significantly associated with community succession dynamics [64]. The aforementioned two points may help explain why the C3 fraction acts as a key factor shaping the composition of the biofilm bacterial community harvested in winter. In summer, tryptophan proteins, aromaticity, and molecular weight were significantly associated with bacterial community (Figure 6c,d). The aromaticity and molecular weight of biofilm WSOM were remarkably lower in summer compared to winter (*p* < 0.01 and *p* < 0.001) (Table S4). We speculate that the seasonally variable biofilm WSOM properties may determine their distinct capacities in shaping biofilm bacterial community composition. The close association of tryptophan proteins with microbial activity has been reported in previous studies [6,47]. At this stage of understanding, we believe that certain factors, e.g., molecular weight and aromaticity in biofilm WSOM, might govern the bacteria community structure, regardless of the sampling seasons.

It is worth noting that FI values showed a significant correlation with changes in bacterial community composition in both seasons, although FI values did not show signal variations among seasons and areas. The results provide additional evidence that the microbial-derived organic matter of biofilm WSOM remains largely stable [65]. The value of FI has been strongly correlated to the relative contribution of microbial-derived versus plant-derived organic matter. The FI values of all samples ranged from 2.40 to 3.04, indicating that biofilm WSOM predominantly originated from microbial activity [66]. It can be assumed that microbial-derived organic matter produced by microbial metabolism occupies an important position in biofilms, especially in the process of shaping microbial communities.

### 4.5. Co-Occurrence Patterns Relate to Seasonal and Spatial Variation

The interactions between microbial communities in turn affect their adaptation to external environmental changes, and the co-occurrence networks constructed based on different areas can reveal the ecological interactions between biofilm bacterial communities [25]. In different areas of the network (Figure S4), the average path distance followed the sequence: Upstream group < Discharge area group < Downstream group (Table S8), reflecting more efficient information processing and material transfer among bacteria influenced by discharge [27]. The value of modularity was highest in the Upstream group and lowest in the Discharge area group, demonstrating that microbial interactions are stronger in the Upstream group and effluent discharge disrupts original interspecies interactions of bacteria, as in [67]. Compared to the Upstream group, the networks from the Discharge area and Downstream groups had fewer nodes and edges, and the average degree decreased along the effluent-receiving river, demonstrating that effluent discharge simplified the network pattern of benthic biofilms [48]. Previous studies have suggested that a more connected network could improve the efficient utilization of carbon and that a highly connected network may also provide more functional redundancy [68,69]. Effluent contains plentiful carbon, nitrogen and some toxic substances, which might possibly limit the complexity of microbial co-occurrence networks in the Discharge area and the Downstream groups. Consequently, effluent discharge may potentially diminish the stability and disturbance resistance of benthic biofilm communities.

Positive correlations in Figure 7 suggest that the interactions are chiefly symbiotic or mutually beneficial. The microbe-organic networks in different seasons were dominated by positive correlations. Generally speaking, a more positive microbe-organic correlation is beneficial for the degradation of refractory substances [70]. Proteobacteria, one of the dominant phyla in the network, can degrade humic substances and tend to form filamentous structures, facilitating the growth of such bacteria in biofilms [71]. Bacteroidetes, another dominant phylum, generally hold special importance for benthic biofilms and some members of these phylum can degrade biopolymers, contributing to the high-molecular-weight fraction of organic matters [72].

Moreover, modules can be considered as functional units, in which the same functional unit can perform the same ecological function with a high degree of in-connection between microbial communities within the same module [73]. Here, bacterial communities and biofilm WSOM components formed a strong network of modules, with a total of six modules in winter and seven modules in summer (Figure 7). By taking advantage of the key modules, we can acquire more information on the interactions between bacterial communities and fluorescent compounds. For example, in Module III of the network derived from samples collected in winter, genus *Kineosporiaceae* was able to convert cellulose and glucose to acetate, butyric acid, and carbon dioxide under anoxic conditions [74]. Genus *Lacihabitans* was proven capable of degrading a multitude of organic compounds including cellulose, chitin, and starch [75]. Similarly, in the network derived from samples collected in summer, genus *Nocardioidaceae* in Module IV was capable of depredating toxic pollutants, alkanes, crude oil, and derivatives [76]. The family *Beijerinckiaceae* is able to fix nitrogen and metabolize carbon. As a consequence, the pollutant degradation capacity of benthic biofilm bacterial communities in summer may also be amended in response to effluent discharge [77]. Genus *Aeromans* is an important pathogenic agent for fish and is harmful to aquatic ecosystems [78]. These findings support the notion that carbon metabolism remains a key ecological function of benthic biofilms in effluent-receiving rivers, and we speculate that there is an enhancement of the degradation of toxic pollutants in summer. Meanwhile, the production of pathogenic bacteria needs to be guarded against. Note that due to the lack of controlled experiments, these results need to be treated with caution. Simulation experiments need to be conducted to clarify the ecological impact of effluent discharge on effluent-receiving rivers in future work.

## 5. Conclusions

In highly urbanized areas, river benthic biofilms are pioneering microbial aggregates responding to effluent discharge from WWTPs. Our study reveals the optical properties of benthic biofilms WSOM in a representative effluent-receiving river. The diversity, function, and assembly of bacterial communities and their co-occurrence patterns were also investigated. After receiving effluent, WSOM in benthic biofilms showed weak humic character and strong autotrophic components. In the Discharge area, the fluorescence characteristics of CDOM and bacterial community diversity exhibited a signal alteration. Both the interspecies interaction of bacteria and the fluorescent nature of biofilm WSOM gradually recover to the conditions exhibited when less affected by effluent discharge. Species turnover was the main factor governing the formation of biofilm diversity patterns. Functional predictions showed that amino acid metabolism and carbohydrate metabolism increased significantly after receiving effluent discharge. Additionally, amino acid-like and humic-like intermediates and tryptophan proteins were found to be key factors affecting bacterial community composition in winter and summer, respectively. The key ecological functions present in the benthic biofilm in the effluent-receiving river were further elucidated by combining the key modules in co-occurrence networks. Future studies will be performed with a focus on WWTPs with different effluent standards to demonstrate the universality of benthic biofilms as an indicator of the ecological response of effluent-receiving rivers.

## Data Availability

The data that support the findings of this study are available from the corresponding author, upon reasonable request.

## Supplementary Materials

The following supporting information can be downloaded at: www.mdpi.com/article/10.3390/ijerph19041994/s1: Figure S1: Taxonomic composition and relative abundance of bacterial communities at the phylum level. Proteobacteria is identified to class level; Figure S2: Mechanisms of microbial community assembly based on stochastic and deterministic models for different (a) seasons and (b) areas; Figure S3: Abundance of various predictive function samples on KEGG pathway level 2 based on PICRUSt. “S-” indicates sampling in summer and “W-” indicates sampling in winter; Figure S4: Co-occurrence network of bacterial communities in (a) Upstream group, (b) Discharge area group, and (c) Downstream group. The node colors represent major modules. The modularity for each network is shown below the figures; Table S1: Physicochemical parameters of water samples at each sampling site; Table S2: Normalized water-soluble organic carbon concentration in biofilm WSOM at varying sampling sites; Table S3: Description of UV-vis absorption spectrum parameters and EEMs parameters; Table S4: UV-visible spectral-derived parameters of biofilm WSOM; Table S5: The discrepancy of the spatial turnover and nestedness pattern indices in different areas and seasons; Table S6: The discrepancy of the spatial turnover and nestedness pattern indices in different areas (resampling); Table S7: Statistics of topological characteristics of empirical and random networks in different seasons; Table S8: Topological properties of benthic biofilm bacterial community networks in different areas [79,80,81].

## References

[1] Kamjunke N., Hertkorn N., Harir M., Schmitt-Kopplin P., Griebler C., Brauns M., von Tumpling W., Weitere M., Herzsprung P. (2019). Molecular change of dissolved organic matter and patterns of bacterial activity in a stream along a land-use gradient. Water Res..

[2] Burdon F.J., Bai Y., Reyes M., Tamminen M., Staudacher P., Mangold S., Singer H., Rasanen K., Joss A., Tiegs S.D. (2020). Stream microbial communities and ecosystem functioning show complex responses to multiple stressors in wastewater. Glob. Chang. Biol..

[3] Waiser M.J., Tumber V., Holm J. (2011). Effluent-dominated streams. Part 1: Presence and effects of excess nitrogen and phosphorus in Wascana Creek, Saskatchewan, Canada. Environ. Toxicol. Chem..

[4] Freixa A., Perujo N., Langenheder S., Romani A.M. (2020). River biofilms adapted to anthropogenic disturbances are more resistant to WWTP inputs. FEMS Microbiol. Ecol..

[5] Drury B., Rosi-Marshall E., Kelly J.J. (2013). Wastewater treatment effluent reduces the abundance and diversity of benthic bacterial communities in urban and suburban rivers. Appl. Environ. Microbiol..

[6] Yu M., Liu S., Li G., Zhang H., Xi B., Tian Z., Zhang Y., He X. (2020). Municipal wastewater effluent influences dissolved organic matter quality and microbial community composition in an urbanized stream. Sci. Total. Environ..

[7] Pereda O., Solagaistua L., Atristain M., de Guzman I., Larranaga A., von Schiller D., Elosegi A. (2020). Impact of wastewater effluent pollution on stream functioning: A whole-ecosystem manipulation experiment. Environ. Pollut..

[8] Romero F., Acuna V., Font C., Freixa A., Sabater S. (2019). Effects of multiple stressors on river biofilms depend on the time scale. Sci. Rep..

[9] Osorio V., Proia L., Ricart M., Perez S., Ginebreda A., Cortina J.L., Sabater S., Barcelo D. (2014). Hydrological variation modulates pharmaceutical levels and biofilm responses in a Mediterranean river. Sci. Total. Environ..

[10] Sabater-Liesa L., Montemurro N., Font C., Ginebreda A., Gonzalez-Trujillo J.D., Mingorance N., Perez S., Barcelo D. (2019). The response patterns of stream biofilms to urban sewage change with exposure time and dilution. Sci. Total. Environ..

[11] Pereda O., von Schiller D., Garcia-Baquero G., Mor J.R., Acuna V., Sabater S., Elosegi A. (2021). Combined effects of urban pollution and hydrological stress on ecosystem functions of Mediterranean streams. Sci. Total. Environ..

[12] Battin T.J., Besemer K., Bengtsson M.M., Romani A.M., Packmann A.I. (2016). The ecology and biogeochemistry of stream biofilms. Nat. Rev. Microbiol..

[13] Besemer K. (2015). Biodiversity, community structure and function of biofilms in stream ecosystems. Res. Microbiol..

[14] Risse-Buhl U., Anlanger C., Kalla K., Neu T.R., Noss C., Lorke A., Weitere M. (2017). The role of hydrodynamics in shaping the composition and architecture of epilithic biofilms in fluvial ecosystems. Water Res..

[15] Sabater S., Guasch H., Ricart M., Romani A., Vidal G., Klunder C., Schmitt-Jansen M. (2007). Monitoring the effect of chemicals on biological communities. The biofilm as an interface. Anal. Bioanal. Chem..

[16] Pu Y., Ngan W.Y., Yao Y., Habimana O. (2019). Could benthic biofilm analyses be used as a reliable proxy for freshwater environmental health?. Environ. Pollut..

[17] Hobbs W.O., Collyard S.A., Larson C., Carey A.J., O’Neill S.M. (2019). Toxic Burdens of Freshwater Biofilms and Use as a Source Tracking Tool in Rivers and Streams. Environ. Sci. Technol..

[18] Tlili A., Corcoll N., Arrhenius Å., Backhaus T., Hollender J., Creusot N., Wagner B., Behra R. (2020). Tolerance Patterns in Stream Biofilms Link Complex Chemical Pollution to Ecological Impacts. Environ. Sci. Technol..

[19] Qiu L., Cui H., Wu J., Wang B., Zhao Y., Li J., Jia L., Wei Z. (2016). Snowmelt-driven changes in dissolved organic matter and bacterioplankton communities in the Heilongjiang watershed of China. Sci. Total. Environ..

[20] Kamjunke N., Herzsprung P., Neu T.R. (2015). Quality of dissolved organic matter affects planktonic but not biofilm bacterial production in streams. Sci. Total. Environ..

[21] Logue J.B., Stedmon C.A., Kellerman A.M., Nielsen N.J., Andersson A.F., Laudon H., Lindstrom E.S., Kritzberg E.S. (2016). Experimental insights into the importance of aquatic bacterial community composition to the degradation of dissolved organic matter. ISME J..

[22] Avila M.P., Brandao L.P.M., Brighenti L.S., Tonetta D., Reis M.P., Staehr P.A., Asmala E., Amado A.M., Barbosa F.A.R., Bezerra-Neto J.F. (2019). Linking shifts in bacterial community with changes in dissolved organic matter pool in a tropical lake. Sci. Total. Environ..

[23] Zhang L., Fang W., Li X., Lu W., Li J. (2020). Strong linkages between dissolved organic matter and the aquatic bacterial community in an urban river. Water Res..

[24] Niu L., Li Y., Li Y., Hu Q., Wang C.D., Hu J., Zhang W., Wang L., Zhang C., Zhang H. (2021). New insights into the vertical distribution and microbial degradation of microplastics in urban river sediments. Water Res..

[25] Zhao Q., Chu S.S., He D., Wu D.M., Mo Q.F., Zeng S.C. (2021). Sewage sludge application alters the composition and co-occurrence pattern of the soil bacterial community in southern China forestlands. Appl. Soil Ecol..

[26] Zhou H., Gao Y., Jia X.H., Wang M.M., Ding J.J., Cheng L., Bao F., Wu B. (2020). Network analysis reveals the strengthening of microbial interaction in biological soil crust development in the Mu Us Sandy Land, northwestern China. Soil Biol. Biochem..

[27] Dong Y., Gao J., Wu Q., Ai Y., Huang Y., Wei W., Sun S., Weng Q. (2020). Co-occurrence pattern and function prediction of bacterial community in Karst cave. BMC Microbiol..

[28] Fang D., Hao L., Cao Z., Huang X.L., Qin M.S., Hu J.C., Liu Y.Q., Sun G. (2020). Combined effects of urbanization and climate change on watershed evapotranspiration at multiple spatial scales. J. Hydrol..

[29] APHA (2005). Standard Methods for the Examination of Water and Wastewater.

[30] Wang J., Meier S., Soininen J., Casamayor E.O., Pan F., Tang X., Yang X., Zhang Y., Wu Q., Zhou J. (2017). Regional and global elevational patterns of microbial species richness and evenness. Ecography.

[31] Chantigny M.H., Harrison-Kirk T., Curtin D., Beare M. (2014). Temperature and duration of extraction affect the biochemical composition of soil water-extractable organic matter. Soil Biol. Biochem..

[32] Huang M., Chai L., Jiang D., Zhang M., Jia W., Huang Y. (2021). Spatial Patterns of Soil Fungal Communities Are Driven by Dissolved Organic Matter (DOM) Quality in Semi-Arid Regions. Microb Ecol..

[33] Stedmon C.A., Bro R. (2008). Characterizing dissolved organic matter fluorescence with parallel factor analysis: A tutorial. Limnol. Oceanogr-Meth..

[34] Bolyen E., Rideout J.R., Dillon M.R., Bokulich N.A., Abnet C.C., Al-Ghalith G.A., Alexander H., Alm E.J., Arumugam M., Asnicar F. (2019). Author Correction: Reproducible, interactive, scalable and extensible microbiome data science using QIIME 2. Nat. Biotechnol..

[35] Callahan B.J., McMurdie P.J., Rosen M.J., Han A.W., Johnson A.J., Holmes S.P. (2016). DADA2: High-resolution sample inference from Illumina amplicon data. Nat. Methods.

[36] Langille M.G., Zaneveld J., Caporaso J.G., McDonald D., Knights D., Reyes J.A., Clemente J.C., Burkepile D.E., Vega Thurber R.L., Knight R. (2013). Predictive functional profiling of microbial communities using 16S rRNA marker gene sequences. Nat. Biotechnol..

[37] Baselga A. (2010). Partitioning the turnover and nestedness components of beta diversity. Global Ecol. Biogeogr..

[38] Stegen J.C., Lin X., Konopka A.E., Fredrickson J.K. (2012). Stochastic and deterministic assembly processes in subsurface microbial communities. ISME J..

[39] Stegen J.C., Lin X., Fredrickson J.K., Chen X., Kennedy D.W., Murray C.J., Rockhold M.L., Konopka A. (2013). Quantifying community assembly processes and identifying features that impose them. ISME J..

[40] Zhou J., Deng Y., Luo F., He Z., Tu Q., Zhi X. (2010). Functional molecular ecological networks. MBio.

[41] Deng Y., Jiang Y.H., Yang Y., He Z., Luo F., Zhou J. (2012). Molecular ecological network analyses. BMC Bioinformatics.

[42] Hu Y., Bai C., Cai J., Dai J., Shao K., Tang X., Gao G. (2018). Co-occurrence network reveals the higher fragmentation of the bacterial community in Kaidu River than its tributaries in Northwestern China. Microbes Environ..

[43] Kothawala D.N., von Wachenfeldt E., Koehler B., Tranvik L.J. (2012). Selective loss and preservation of lake water dissolved organic matter fluorescence during long-term dark incubations. Sci. Total Environ..

[44] Osburn C.L., Wigdahl C.R., Fritz S.C., Saros J.E. (2011). Dissolved organic matter composition and photoreactivity in prairie lakes of the U.S. Great Plains. Limnol. Oceanogr..

[45] Retelletti Brogi S., Jung J.Y., Ha S.Y., Hur J. (2019). Seasonal differences in dissolved organic matter properties and sources in an Arctic fjord: Implications for future conditions. Sci. Total Environ..

[46] Jørgensen L., Stedmon C.A., Kragh T., Markager S., Middelboe M., Søndergaard M. (2011). Global trends in the fluorescence characteristics and distribution of marine dissolved organic matter. Marine Chemistry.

[47] Hambly A.C., Arvin E., Pedersen L.F., Pedersen P.B., Seredynska-Sobecka B., Stedmon C.A. (2015). Characterising organic matter in recirculating aquaculture systems with fluorescence EEM spectroscopy. Water Res..

[48] Fan K., Weisenhorn P., Gilbert J.A., Chu H. (2018). Wheat rhizosphere harbors a less complex and more stable microbial co-occurrence pattern than bulk soil. Soil Biol. Biochem..

[49] Wang M., Chen Y. (2018). Generation and characterization of DOM in wastewater treatment processes. Chemosphere.

[50] Simsek H., Wadhawan T., Khan E. (2013). Overlapping photodegradable and biodegradable organic nitrogen in wastewater effluents. Environ. Sci. Technol..

[51] Li J., Wang L., Geng J., Li S., Yu Q., Xu K., Ren H. (2020). Distribution and removal of fluorescent dissolved organic matter in 15 municipal wastewater treatment plants in China. Chemosphere.

[52] Wang Y., Hu Y., Yang C., Wang Q., Jiang D. (2019). Variations of DOM quantity and compositions along WWTPs-river-lake continuum: Implications for watershed environmental management. Chemosphere.

[53] Maqbool T., Zhang J., Qin Y., Ly Q.V., Asif M.B., Zhang X., Zhang Z. (2020). Seasonal occurrence of N-nitrosamines and their association with dissolved organic matter in full-scale drinking water systems: Determination by LC-MS and EEM-PARAFAC. Water Res..

[54] Zhang L., Fang W., Li X., Gao G., Jiang J. (2020). Linking bacterial community shifts with changes in the dissolved organic matter pool in a eutrophic lake. Sci. Total Environ..

[55] Yang X., Zhou Z., Raju M.N., Cai X., Meng F. (2017). Selective elimination of chromophoric and fluorescent dissolved organic matter in a full-scale municipal wastewater treatment plant. J. Environ. Sci..

[56] Perujo N., Freixa A., Vivas Z., Gallegos A.M., Butturini A., Romaní A.M. (2015). Fluvial biofilms from upper and lower river reaches respond differently to wastewater treatment plant inputs. Hydrobiologia.

[57] Pascual-Benito M., Balleste E., Monleon-Getino T., Urmeneta J., Blanch A.R., Garcia-Aljaro C., Lucena F. (2020). Impact of treated sewage effluent on the bacterial community composition in an intermittent mediterranean stream. Environ. Pollut..

[58] Zhang W., Lei M., Li Y., Wang P., Wang C., Gao Y., Wu H., Xu C., Niu L., Wang L. (2019). Determination of vertical and horizontal assemblage drivers of bacterial community in a heavily polluted urban river. Water Res..

[59] Brislawn C.J., Graham E.B., Dana K., Ihardt P., Fansler S.J., Chrisler W.B., Cliff J.B., Stegen J.C., Moran J.J., Bernstein H.C. (2019). Forfeiting the priority effect: Turnover defines biofilm community succession. ISME J..

[60] Xu R., Zhang S., Meng F. (2020). Large-sized planktonic bioaggregates possess high biofilm formation potentials: Bacterial succession and assembly in the biofilm metacommunity. Water Res..

[61] Li Y., Xu C., Zhang W., Lin L., Wang L., Niu L., Zhang H., Wang P., Wang C. (2020). Response of bacterial community in composition and function to the various DOM at river confluences in the urban area. Water Res..

[62] Neis E.P., Dejong C.H., Rensen S.S. (2015). The role of microbial amino acid metabolism in host metabolism. Nutrients.

[63] Cabrera J.M., Garcia P.E., Pedrozo F.L., Queimalinos C.P. (2020). Dynamics of the dissolved organic matter in a stream-lake system within an extremely acid to neutral pH range: Agrio-Caviahue watershed. Spectrochim. Acta. A. Mol. Biomol. Spectrosc..

[64] Zhou L., Zhou Y., Tang X., Zhang Y., Jang K.S., Szekely A.J., Jeppesen E. (2021). Resource aromaticity affects bacterial community successions in response to different sources of dissolved organic matter. Water Res..

[65] Wu K., Lee T.H., Chen Y.L., Wang Y.S., Wang P.H., Yu C.P., Chu K.H., Chiang Y.R. (2019). Metabolites Involved in Aerobic Degradation of the A and B Rings of Estrogen. Appl. Environ. Microb..

[66] Zhang P., Cao C., Wang Y.H., Yu K., Liu C., He C., Shi Q., Wang J.J. (2021). Chemodiversity of water-extractable organic matter in sediment columns of a polluted urban river in South China. Sci. Total Environ..

[67] Lu Y., Zhang W., Li Y., Zhang C., Wang L., Niu L., Zhang H. (2021). Microbial community shift via black carbon: Insight into biological nitrogen removal from microbial assemblage and functional patterns. Environ. Res..

[68] Morrien E., Hannula S.E., Snoek L.B., Helmsing N.R., Zweers H., de Hollander M., Soto R.L., Bouffaud M.L., Buee M., Dimmers W. (2017). Soil networks become more connected and take up more carbon as nature restoration progresses. Nat. Commun..

[69] Yang W., Jing X., Guan Y., Zhai C., Wang T., Shi D., Sun W., Gu S. (2019). Response of Fungal Communities and Co-occurrence Network Patterns to Compost Amendment in Black Soil of Northeast China. Front. Microbiol..

[70] Zhu P., Li Y., Gao Y., Yin M., Wu Y., Liu L., Du N., Liu J., Yu X., Wang L. (2021). Insight into the effect of nitrogen-rich substrates on the community structure and the co-occurrence network of thermophiles during lignocellulose-based composting. Bioresour. Technol..

[71] Newton R.J., Jones S.E., Eiler A., McMahon K.D., Bertilsson S. (2011). A guide to the natural history of freshwater lake bacteria. Microbiol. Mol. Biol. Rev..

[72] Kirchman D.L. (2002). The ecology of Cytophaga-Flavobacteria in aquatic environments. FEMS Microbiol. Ecol..

[73] Yang J., Jiang H., Liu W., Huang L., Huang J., Wang B., Dong H., Chu R.K., Tolic N. (2020). Potential utilization of terrestrially derived dissolved organic matter by aquatic microbial communities in saline lakes. ISME J..

[74] Schellenberger S., Kolb S., Drake H.L. (2009). Metabolic responses of novel cellulolytic and saccharolytic agricultural soil Bacteria to oxygen. Environ. Microbiol..

[75] Joung Y., Kim H., Kang H., Lee B.I., Ahn T.S., Joh K. (2014). Lacihabitans soyangensis gen. nov.; sp. nov.; a new member of the family Cytophagaceae, isolated from a freshwater reservoir. Int. J. Syst. Evol. Microbiol..

[76] Zhou J., Deng Y., Luo F., He Z., Yang Y. (2011). Phylogenetic molecular ecological network of soil microbial communities in response to elevated CO_2_. mBio.

[77] Dedysh S.N., Haupt E.S., Dunfield P.F. (2016). Emended description of the family Beijerinckiaceae and transfer of the genera Chelatococcus and Camelimonas to the family Chelatococcaceae fam. nov. Int. J. Syst. Evol. Microbiol..

[78] Janda J.M., Abbott S.L. (2010). The Genus Aeromonas: Taxonomy, Pathogenicity, and Infection. Clin. Microbiol. Rev..

[79] Zepp R.G., Sheldon W.M., Moran M.A. (2004). Dissolved organic fluorophores in southeastern US coastal waters: Correction method for eliminating Rayleigh and Raman scattering peaks in excitation–emission matrices. Mar. Chem..

[80] Lee M.H., Lee Y.K., Derrien M., Choi K., Shin K.H., Jang K.S., Hur J. (2019). Evaluating the contributions of different organic matter sources to urban river water during a storm event via optical indices and molecular composition. Water Res..

[81] Ye Q.H., Wang Y.H., Zhang Z.T., Huang W.L., Li L.P., Li J.T., Liu J.S., Zheng Y., Mo J.M., Zhang W. (2020). Dissolved organic matter characteristics in soils of tropical legume and non-legume tree plantations. Soil Biol. Biochem..

