# Supplementary material for: Benthic Biofilm Bacterial Communities and Their Linkage with Water-Soluble Organic Matter in Effluent Receivers
Source: Int J Environ Res Public Health. 2022 Feb 10;19(4):1994. doi: 10.3390/ijerph19041994 (PMC8872271; doi:10.3390/ijerph19041994)
Supplement: Supplementary file 1 [file ijerph-19-01994-s001.zip › ijerph-1578316-supplementary.pdf]

International Journal of
Environmental Research and Public Health

MDPI

Supplementary Materials

Biofilm collection method

Samples were only collected from stones and rocks from riffle/run area of the river at each site. Separated cobble-sized rocks exposed to varying light conditions were selected. A sterile scraper and a toothbrush were used to scrape and brush the biofilm attached on the rocks carefully and composited in a stainless-steel container. Use the scraper to scrape biofilm from the upper surface of rocks into the sample jar. Use the toothbrush to loosen remaining periphyton. Approximately 30 g of biofilm in wet weight was gathered from each site. All samples were stored in a sample storage box in ice-water mixture and dark condition during their transport to laboratory.

Spectral analysis

UV-vis absorption spectra: The absorbance was measured in the range of 200~600 nm with a scan interval of 1 nm, and Milli-Q water was used as a blank.

The excitation-emission matrices: The CDOM in WSOM were using a 1-cm quartz cuvette, and picked 150w xenon arc lamp as the excitation source of the photometer. The following parameters were set for all samples: excitation (Ex) and emission (Em) wavelengths from 200 to 600 nm with an interval of 2 nm and a scan rate of 30,000 nm/min. Milli-Q water was used as a blank control. Based on the original EEM, we performed blank subtraction, removal of internal filtering effects and first-order/second-order Riley and Raman scattering correction operations [1].

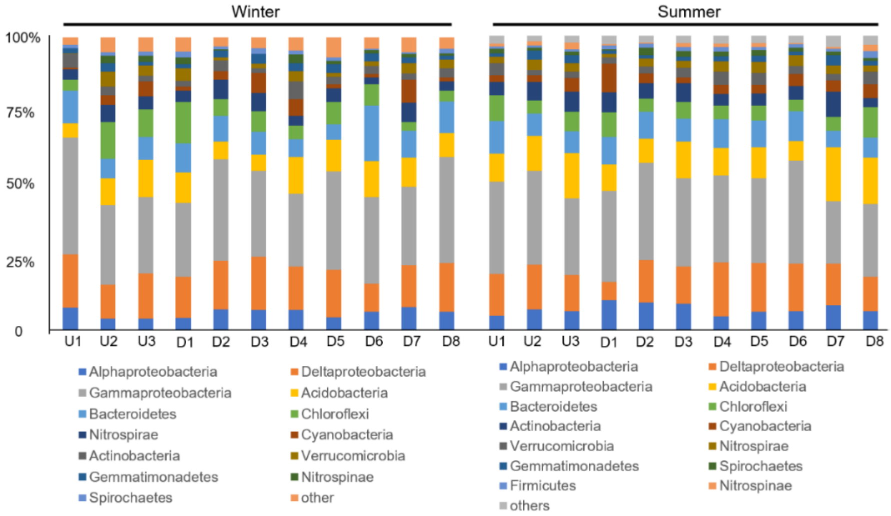

Figure S1. Taxonomic composition and relative abundance of bacterial communities at the phylum level. Proteobacteria is identified to class level.

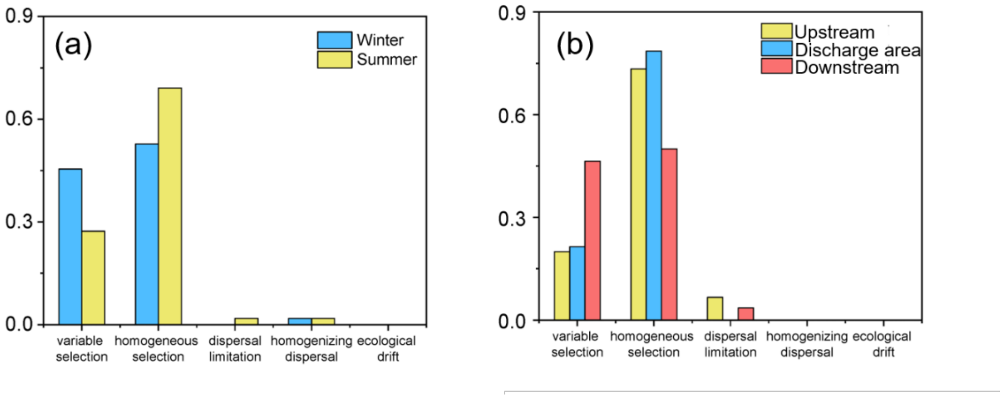

Figure S2. Mechanisms of microbial community assembly based on stochastic and deterministic models for different (**a**) seasons and (**b**) areas.

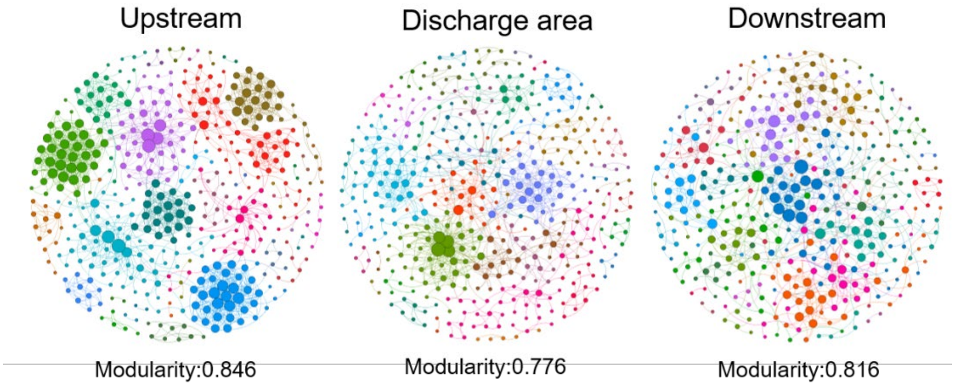

Figure S3. Cooccurrence network of bacterial communities in (a) Upstream group, (b) Discharge area group and (c) Downstream group. The node colors represent major modules. The modularity for each network is shown below the figures.

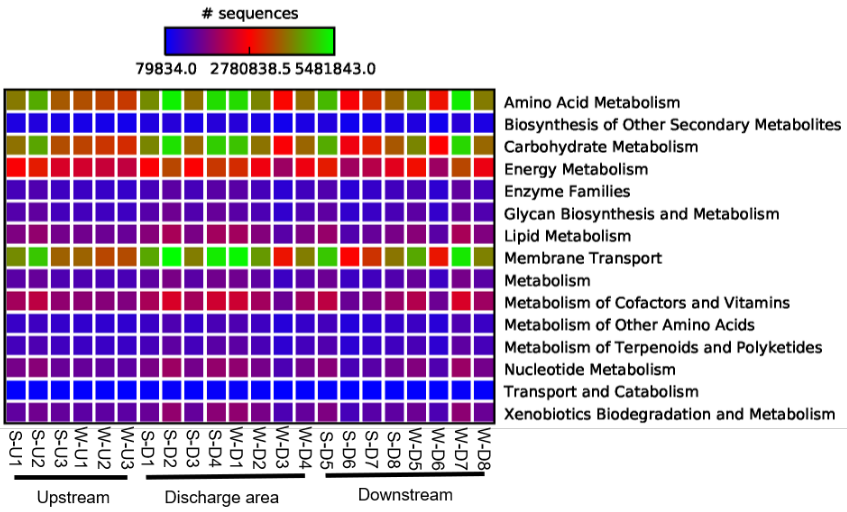

Figure S4. Abundance of various predictive functions sample on KEGG pathway level 2 based on PICRUSt. "S-" indicates sampling in summer and "W-" indicates sampling in winter.

Table S1. Physicochemical parameters of water samples at each sampling site.

Areas	Upstream group			Discharge area group				Downstream group			
Sampling sites	U1	U2	U3	D1	D2	D3	D4	D5	D6	D7	D8
Season	Summer										
DO (mg/L)	3.80	3.20	4.00	4.70	4.20	4.00	6.80	5.00	4.20	4.60	3.90
pH	7.05	7.11	7.08	6.87	7.02	7.12	7.28	7.25	7.24	7.27	7.20
T (°C)	22.4	23.6	24.50	27.90	28.70	27.60	28.00	28.30	28.00	28.10	28.00
TN (mg/L)	12.80	22.50	17.52	20.50	14.70	17.20	12.80	8.16	8.67	9.09	9.54
TP (mg/L)	0.16	0.23	0.18	0.25	0.15	0.10	0.10	0.13	0.14	0.12	0.15
Season	Winter										
DO (mg/L)	8.75	8.66	8.50	8.36	8.00	8.66	7.13	7.81	8.20	8.10	8.28
pH	7.58	7.50	7.25	7.49	7.50	7.54	7.54	7.50	7.65	7.61	7.64
T (°C)	10.70	10.50	10.30	10.10	10.60	11.00	9.30	10.30	10.30	10.40	10.10
TN (mg/L)	9.20	8.87	10.56	13.40	12.03	15.35	13.44	10.17	9.95	7.67	8.38
TP (mg/L)	0.08	0.15	0.13	0.22	0.35	0.16	0.07	0.13	0.11	0.07	0.11

The water level at the time of sampling in summer was 8.48 m; in winter is 6.42 m.

Table S2. Normalized water-soluble organic carbon concentration in biofilm WSOM at varying sampling sites.

Areas	Sampling site	DOC (mg C/g) Summer	DOC (mg C/g) Winter
Upstream group	U1	201.6±8.2	151.0±3.8
	U2	181.8±6.5	160.7±6.6
	U3	213.6±4.4	144.1±3.9
Discharge area group	D1	331.2±8.4	208.3±5.1
	D2	278.4±7.9	172.8±6.8
	D3	240.6±4.5	141.4±3.2
	D4	268.2±6.9	150.5±4.0
Downstream group	D5	268.6±3.3	325.8±5.5
	D6	232.8±4.9	263.0±4.6
	D7	228.6±4.2	352.7±3.6
	D8	338.4±6.7	360.4±8.4

Table S3. Description of UV-vis absorption spectrum parameters and EEMs parameters.

Index	Formula	Environment significance	Reference
$SUVA_{254}$	$SUVA_{254} = a_{(254)}/DOC$	Absorption coefficient at 254 nm wavelength, normalized using DOC concentration. Used to characterize the aromatic properties of WOSM.	[2]
E_{254}/E_{204}	$E_{254}/E_{204} = a_{(254)}/a_{(204)}$	Ratio of absorption coefficients at wavelengths of 254 nm and 204 nm. Used to characterize the hydrophilicity of WSOM.	[3]
S_R	$a(\lambda) = a(\lambda_0)\exp[S(\lambda_0-\lambda)]$ $S_R = S_{(275\text{-}295)}/S_{(350\text{-}400)}$	Ratio of the slope of the natural logarithmic fit line for absorbance at 275-295 nm and 350-400 nm. Used to characterize the molecular weight of WSOM.	[3]
Fluorescence index (FI)	$FI = \frac{Em=470nm,Ex=370nm}{Em=520nm,Ex=370nm}$	At an excitation wavelength of 370 nm, the ratio of the emission intensity at 470 nm to at 520 nm. Used to characterize the source of humic substances in WSOM (from terrestrial sources or microbial sources).	[2,4]
Biological index (BIX)	$BIX = \frac{Em=380nm,Ex=310nm}{max(Em=400nm-435nm),Ex=310nm}$	The ratio of emission intensity at 380 nm to the maximum intensity observed within the emission range of 420–435 nm at an excitation wavelength of 310 nm. Used to characterize the proportion of newly produced autochthonous in WSOM.	[2,5]
Humification index (HIX)	$HIX = \frac{\int(Em=435nm-480nm),Ex=254nm}{\int(Em=300nm-345nm),Ex=254nm}$	Calculated by the peak area under the emission spectra at 435–480 nm divided by that within the emission range of 300–345 nm, at an excitation wavelength of 254 nm. Used to characterize the degree of humification in WSOM.	[2,5]

Table S4. UV-visible spectral-derived parameters of biofilms WSOM.

Areas		Upstream group				Discharge area group					Downstream group				
Sampling sites		B1	B2	B3	mean	A1	A2	A3	A4	mean	A5	A6	A7	A8	mean
Parameter															
$SUVA_{254}$	Winter	4.37	4.10	3.81	**4.09**	4.71	2.45	2.19	1.86	**2.80**	2.31	2.39	2.15	2.27	**2.28**
	Summer	1.55	1.01	1.33	**1.30**	4.06	2.26	2.50	1.15	**2.49**	1.65	1.63	2.29	1.66	**1.81**
E_{254}/E_{204}	Winter	0.16	0.25	0.20	**0.20**	0.29	0.25	0.23	0.11	**0.22**	0.33	0.31	0.37	0.39	**0.35**
	Summer	0.25	0.22	0.29	**0.25**	0.51	0.34	0.33	0.23	**0.35**	0.25	0.24	0.29	0.24	**0.26**
S_R	Winter	0.36	0.39	0.41	**0.39**	0.60	0.48	0.35	0.55	**0.50**	0.60	0.68	0.75	0.65	**0.67**
	Summer	1.32	1.49	1.43	**1.41**	0.83	1.09	1.34	1.31	**1.14**	1.36	1.27	1.21	1.34	**1.30**

Table S5. The discrepancy of the spatial turnover and nestedness pattern indices in different areas and seasons.

Areas	β_{SOR}	β_{SIM}	β_{NES}
Upstream group	0.76	0.74	0.02
Discharge area group	0.84	0.82	0.02
Downstream group	0.85	0.82	0.03
Mean	0.82	0.79	0.02
Winter	0.86	0.84	0.02
Summer	0.85	0.83	0.02

Table S6. The discrepancy of the spatial turnover and nestedness pattern indices in different areas (resampling).

Areas	β'_{SOR}	β'_{SIM}	β'_{NES}
Upstream group (6)	0.76	0.74	0.02
Discharge area group (8 vs. 6)	0.81	0.8	0.02
Downstream group (8 vs. 6)	0.82	0.79	0.03

Table S7. Statistics of topological characteristics of empirical and random networks in different seasons.

	Empirical Network		Random Network	
	Winter	Summer	Winter	Summer
Modularity	0.479	0.407	0.132±0.009	0.100±0.008
Average clustering coefficient	0.249	0.246	0.056±0.004	0.084±0.005
Average path length	3.328	3.268	2.802±0.011	2.684±0.010
Betweenness	0.025	0.018	0.035±0.004	0.033±0.003
Stress centrality	0.371	0.289	0.247±0.020	0.278±0.020
Eigenvector centrality	0.181	0.170	0.132±0.010	0.128±0.006
Density	0.026	0.030	0.026±0.000	0.030±0.000
Transitivity	0.273	0.343	0.061±0.002	0.096±0.002

Table S8. Topological properties of benthic biofilm bacterial community networks in different areas.

	Upstream group	Discharge area group	Downstream group
Total nodes	450	446	397
Total edges	1510	987	735
Average degree (avgK)	6.711	4.426	3.703
Average path distance (GD)	5.973	6.091	6.503
Connectedness (con)	0.681	0.638	0.709
Number of modules	38	41	33
Modularity	0.846	0.776	0.816

Reference

1. Zepp, R.G.; Sheldon, W.M; Moran, M.A. Dissolved organic fluorophores in southeastern US coastal waters: correction method for eliminating Rayleigh and Raman scattering peaks in excitation–emission matrices. *Mar. Chem.* **2004**, *89*(1–4), 15–36.
2. Lee, M.H.; Lee, Y.K.; Derrien, M.; Choi, K.; Shin, K.H.; Jang, K.S.; Hur, J. Evaluating the contributions of different organic matter sources to urban river water during a storm event via optical indices and molecular composition. *Water Res.* **2019**, *165*, 115006.
3. Yu, M.; Liu, S.; Li, G.; Zhang, H.; Xi, B.; Tian, Z.; Zhang, Y.; He, X. Municipal wastewater effluent influences dissolved organic matter quality and microbial community composition in an urbanized stream. *Sci. Total Environ.* **2020**, *705*, 135952.
4. Li, J.; Wang, L.; Geng, J.; Li, S.; Yu, Q.; Xu, K.; Ren, H. Distribution and removal of fluorescent dissolved organic matter in 15 municipal wastewater treatment plants in China. *Chemosphere* **2020**, *251*, 126375.
5. Ye, Q.H.; Wang, Y.H.; Zhang, Z.T.; Huang, W.L.; Li, L.P.; Li, J.T.; Liu, J.S.; Zheng, Y.; Mo, J.M.; Zhang, W.; Wang, J.J. Dissolved organic matter characteristics in soils of tropical legume and non-legume tree plantations. *Soil Biol. Biochem.* **2020**, *148*, 107880.
